# Breast-cancer detection using blood-based infrared molecular fingerprints

**DOI:** 10.1186/s12885-021-09017-7

**Published:** 2021-12-02

**Authors:** Kosmas V. Kepesidis, Masa Bozic-Iven, Marinus Huber, Nashwa Abdel-Aziz, Sharif Kullab, Ahmed Abdelwarith, Abdulrahman Al Diab, Mohammed Al Ghamdi, Muath Abu Hilal, Mohun R. K. Bahadoor, Abhishake Sharma, Farida Dabouz, Maria Arafah, Abdallah M. Azzeer, Ferenc Krausz, Khalid Alsaleh, Mihaela Zigman, Jean-Marc Nabholtz

**Affiliations:** 1 Department of Laser Physics, Ludwig Maximilian University of Munich (LMU), Garching, Germany; 2 Laboratory for Attosecond Physics, Max Planck Institute of Quantum Optics (MPQ), Garching, Germany; 3 Oncology Centre, King Saud University (Medical City), Riyadh, Saudi Arabia; 4 Clinical Operations, International Cancer Research Group (ICRG), Sharjah, United Arab Emirates; 5 Pathology Department, King Saud University, Riyadh, Saudi Arabia; 6 Physics and Astronomy Department, Attosecond Science Laboratory, King Saud University, Riyadh, Saudi Arabia

**Keywords:** Breast cancer, Infrared spectroscopy, Liquid biopsy

## Abstract

**Background:**

Breast cancer screening is currently predominantly based on mammography, tainted with the occurrence of both false positivity and false negativity, urging for innovative strategies, as effective detection of early-stage breast cancer bears the potential to reduce mortality. Here we report the results of a prospective pilot study on breast cancer detection using blood plasma analyzed by Fourier-transform infrared (FTIR) spectroscopy – a rapid, cost-effective technique with minimal sample volume requirements and potential to aid biomedical diagnostics. FTIR has the capacity to probe health phenotypes via the investigation of the full repertoire of molecular species within a sample at once, within a single measurement in a high-throughput manner. In this study, we take advantage of cross-molecular fingerprinting to probe for breast cancer detection.

**Methods:**

We compare two groups: 26 patients diagnosed with breast cancer to a same-sized group of age-matched healthy, asymptomatic female participants. Training with support-vector machines (SVM), we derive classification models that we test in a repeated 10-fold cross-validation over 10 times. In addition, we investigate spectral information responsible for BC identification using statistical significance testing.

**Results:**

Our models to detect breast cancer achieve an average overall performance of 0.79 in terms of area under the curve (AUC) of the receiver operating characteristic (ROC). In addition, we uncover a relationship between the effect size of the measured infrared fingerprints and the tumor progression.

**Conclusion:**

This pilot study provides the foundation for further extending and evaluating blood-based infrared probing approach as a possible cross-molecular fingerprinting modality to tackle breast cancer detection and thus possibly contribute to the future of cancer screening.

**Supplementary Information:**

The online version contains supplementary material available at 10.1186/s12885-021-09017-7.

## Background

Breast cancer (BC) represents the most frequent cancer in women with a global incidence above 2 million, and an annual mortality above 600,000 patients in 2018 [1, 2]. The cure rate remains correlated with the stage at diagnosis; therefore, early detection and screening programs are crucial [3–6]. Often, BC screening is based upon radiologic approaches, mostly mammography [4]. These screening modalities, predominantly applied in developed countries, are associated with a significant reduction in mortality (19% overall reduction of the relative risk [1]). However, major limitations and debatable cost-effectiveness of these approaches persist [4, 6]. Due to the limited sensitivity and specificity of current medical diagnostics, cancer can either be overlooked (false negatives) or falsely detected (false positives), leading to either delayed interventions or unnecessary, potentially harmful investigations or psychological stress [7]. Also, BC screening in certain regions of the world remains rudimentary despite grim global projections suggesting a doubling of BC cases within the coming 20 years, mostly in these countries [1].

This concerning situation calls for additional strategies for BC screening, as detection of early-stage BC bears potential to significantly reduce mortality. Hence, there is a high need for complementing current medical diagnostics with efficient, non-invasive or minimally-invasive methods that could possibly lead to new easily implementable high-throughput screening and detection approaches, prior to tissue-biopsy-based diagnostics and molecular profiling [8].

Liquid biopsies have attracted interest over the past decade as a non-invasive approach for disease detection, screening and cancer monitoring [9]. Molecular analyses of human blood derivatives, such as plasma or serum, provide systemic molecular information, and enable novel routes of diagnostics [8, 10]. So far, most liquid biopsies predominantly rely on the analysis of a few pre-selected analytes and biomarkers. Although the emergence of highly sensitive and molecule-specific methods in the fields of proteomics [11–13], metabolomics [14, 15], and genomics [16–18] has led to the discovery of thousands of different biomarker candidates, only a few of them have been validated and transferred to the clinic so far [19]. Moreover, given the complexity of the disease as well as its etiology, increasing the number of analytical methods for cancer detection, such as in multi-omics, could potentially lead to higher detection rates at early stage. However, practically, this will lead to unfeasibly high costs for broad clinical use. It is thus evident that methods that have the capacity to capture information across the entire molecular landscape would be advantageous.

Infrared molecular spectroscopy may be very beneficial here − it detects signals from all types of molecules in a sample in a single time- and cost-effective measurement in a label-free manner [20, 21]. When applied to blood plasma (or serum) samples, infrared spectroscopy delivers infrared molecular fingerprints (IMFs) reflecting the chemical composition of a sample, i.e. the person’s molecular blood phenotype [22, 23]. Even though the IMF of molecularly highly complex blood plasma can only partially be traced back to its molecular origin [24], it may be sensitive and specific to the health state of an individual. In a recent longitudinal study, we have shown that defined workflows to collect, store, process and measure human liquid biopsies lead to reproducible IMFs in healthy, non-symptomatic individuals that are stable over clinically relevant time scales [22, 23]. Numerous studies have shown the potential of blood-based IMFs for the detection of breast cancer [25–28]. Despite these promising initial results, the majority of these studies had a high risk of bias due to patient selection [29]. In fact, it was shown that IMFs are susceptible to external confounding factors, such as those related to sample handling and data collection, as well as to inherent biological variations (e.g. age, body-mass index) that can however affect cancer detection [30]. Since many cancer-related therapies may leave footprints in the chemical composition of peripheral blood, it is essential to evaluate the extent of infrared fingerprint differences at the time when cancer patients have only been diagnosed with malignancy, prior to any cancer-related therapy. This has not been assessed previously, and the estimation of a blood-based infrared fingerprinting approach as a new BC screening modality was not evaluated. In this work, we measured intact blood plasma samples, with FTIR transmission spectroscopy directly in liquid form, prior to any cancer-related therapy, along with non-symptomatic reference individuals, which have been carefully matched to BC cases. By applying support vector machine (SVM) algorithms to train models for binary classification, we obtained a detection efficiency of about 0.79 (area un- der the receiver operating characteristic (ROC) curve, AUC). The present study provides a first estimation of feasibility to directly probe liquid blood plasma for minimally-invasive BC detection, an approach that is easily implementable and could be extended to high-throughput BC screening applications.

## Methods

### Study population and sample collection

Presented results are based on a prospective, single center, observational clinical study. The aim of the study was to assess whether the combination of infrared spec- troscopy of liquid biopsies (blood plasma) with machine learning infrared spectral analyses has any capacity to detect breast cancer (BC). For this purpose, a cohort of female patients diagnosed with BC at the Oncology Centre, King Saud Univer- sity Medical City (KSUMC), Riyadh, Saudi Arabia, was compared with a cohort of women without BC, reference individuals. Inclusion criteria for participation in the study were as follows: Asymptomatic reference individuals were adult females participating in organized or voluntary BC screening, assessed with mammography and (if necessary) breast ultrasound and/or magnetic resonance imaging (MRI). Patients with BC were included after confirmation of pathological diagnosis of invasive breast cancer and prior to any therapeutic intervention for breast cancer. Subjects included in the trial were identified by a trial-specific code, guaranteeing their anonymity.

For the purpose of the study up to 19,6 ml of venous blood was collected per enrolled subject. The tubes were centrifuged for 10 min at 7000 g at a temperature of 4 °C and the supernatants of blood plasma were then aliquoted into 1.5 ml tubes (1 ml plasma each) and stored at 80 °C. These procedures were carried out at the KSUMC. The 8 aliquots of each sample were numbered anonymously. The correspondence list between the subject number and the aliquot number were maintained by the clinical research associate (CRA) coordinator at KSUMC. Samples were processed using same standard operating procedures and shipped from the KSUMC to measurement laboratories at the LMU on dry ice. They have all been processed simultaneously, and have all undergone the same number of freeze-thaw cycles. Once all the samples have been collected and stored (from all individuals involved), these have been all defrosted and measured as liquids within the same measurement campaign along the same procedure. Standardization of procedures and workflows applied assured for minimalization of possible noise due to sample preparation as well as facilitated sufficient reproducibility.

The BC patient group (*n* = 26) consisted of patients diagnosed at KSUMC with the following characteristics: mean age: 49 years (30-62), previous pregnancies: 17 patients (65.4%), pre/peri-menopausal: 11 patients (42.3%), operable non-metastatic BC (stage IA-IIIA): 16 patients (61.5%), invasive ductal carcinoma: 24 patients (92.3%), estrogen receptors positive: 14 patients (53.8%) and HER2 positive: 17 patients (65.4%). It is important to note that patients are regularly referred to KSUMC from secondary hospitals where cancer medications are not readily available (e.g. anti-HER2 monoclonal antibodies). Therefore, the breast cancer accrual at KSUMC does not reflect the usual split between breast cancer molecular subtypes and thus leads to, in particular, an excess of HER2-positive molecular subtypes.

Achieving covariate balance between cases and controls is a standard procedure in observational studies for minimizing the effect of confounding factors and limiting the bias throughout all derived results. In this work, we seek balance in terms of age and BMI. This is achieved by pairwise matching. Out of the 67 samples of the initial control group (collected within BC screening programme), given our criteria only 26 individuals of these were selected for inclusion into a control group that is in covariate balance with the collected BC cases. Table 1 shows the characteristics of the balanced cohort, used for further analysis. In addition, a detailed anonymized file (metafile.xlsx) that lists all available information of the recruited individuals (28 potential cases and 67 potential controls, before matching) is provided along with the manuscript.

**Table 1:** Characteristics of the balanced cohort

Covariates	BC cases (*n* = 26)	References (*n* = 26)
Age in years (mean *±* std)	49 *±* 9	44 *±* 7
BMI in kg/m^2^ (mean *±* std)	29 *±* 6	27 *±* 6
Gender (% female)	100	100

### Spectroscopic analysis

**Fig. 1:**
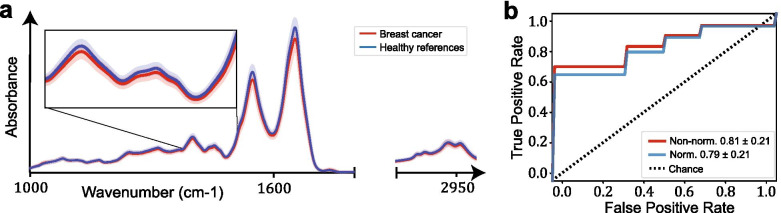
Infrared spectra and classification. **a** Distributions of measured spectra (after water correction) for cases and controls. Solid lines indicate the means of all measurements in each group and shaded areas depict the corresponding standard deviations. **b** Average ROC curves extracted from a repeated 10-fold cross-validation over 10 times for binary classification using linear SVM

The spectroscopic measurements were performed in liquid phase with an automated FTIR device (MIRA-Analyzer, micro-biolytics GmbH) with a flow-through transmission cuvette (CaF2 with 8 μm path length). The spectra were acquired with a resolution of 4 cm^*−* 1^ in a spectral range between 950 cm^*−* 1^ and 3050 cm^*−* 1^. A water reference spectrum was recorded after each sample measurement to reconstruct the IR absorption spectra. To track potential experimental errors throughout the entire experiment [31], a measurement of pooled human plasma (BioWest, Nuailĺe, France) was performed after every 5 samples. Negative values of absorbance, which occurs because the liquid sample contains less water than the reference (pure water), was corrected for by a previously described approach [22]. It is known from measurements of dried plasma that there is no significant absorption in the wavenumber region 2000-2300 cm^*−* 1^, resulting in a flat absorption baseline. This is also confirmed to approximately hold for the case of liquid plasma. We used this fact as a criterion for adding to each spectrum a previously measured water absorption spectrum to account for the missing water in the sample measurement and minimize the average slope in this region in order to obtain a flat baseline. All spectra were truncated to 1000-3000 cm^*−* 1^ and removed the entire silent region (1800-2800 cm^*−* 1^). Finally, to correct for experimental (instrumental/measurement) variations that can affect the overall absorbance of a fingerprint, all spectra were normalized as vectors, using Euclidean (L2) norm. Panel (a) of Fig. 1 shows the distributions of measured spectra (after water correction) of the BC cases and their associated controls. The infrared spectral pre-processing was performed similarly to a previous work [22].

### Data analysis

To derive classification models, we used Scikit-Learn (v. 0.23.2), an open-source machine-learning framework in Python (v.3.7.6). We trained binary-classification models using linear SVM. Performance evaluation was carried out using repeated stratified 10-fold cross-validation and its visualization using the notion of the ROC curve. The results of the cross validation are reported in terms of descriptive statistics: the mean value of the resulting AUC distribution and its standard deviation. For statistically comparing two groups of spectra (i.e. cases, references), we followed three approaches. First, we calculated the “differential fingerprint” (differential infrared spectrum), defined as the difference between the mean absorbance per wavenumber of the cases a contrasted against the standard deviation of the reference group for obtaining a visual understanding of which wavenumbers are potentially useful for distinguishing/classifying the two populations. Such a graph serves as a visual representation of what is known as the “effect size” [32], which can be obtained by standardizing the differential fingerprint and has an evident relation to the AUC per wavenumber. Secondly, we performed t-test (testing the hypothesis that two populations have equal means) for extracting two-tailed *p*-values per wavenumber. As a last, third step, we make use of the Mann–Whitney U test (also known as Wilcoxon rank-sum test) for extracting the U statistic and calculating the AUC per wavenumber by the relation *AUC* = *U/*(*n*_1_ *× n*_2_), where *n*_1_ and *n*_2_ are the sizes of the two groups.

## Results

### Infrared molecular fingerprinting for classification of breast cancer

To evaluate whether IMF probing of liquid plasma has any capacity to detect BC, we performed binary classification for distinction between the BC patients and the matched asymptomatic reference individuals (Table 1 and Fig. 1a). The detection efficiencies achieved on the test sets correspond to an AUC value of 0.79 for normalized FTIR spectra. A higher AUC value of 0.81 could be achieved using non-normalized spectra (Fig. 1b). Despite the higher AUC obtained for non- normalized spectra, we consider the analysis of normalized data to be more reliable. Vector normalization reduces measurement uncertainty which can be a major factor of bias, especially in cases of small sample sizes. Overall, these results deliver the first evidence that the molecular differences between reference individuals and matched therapy-naive BC patient females can be detected with infrared fingerprinting of fluid blood plasma.

### Infrared spectral probing of breast cancer

**Fig. 2:**
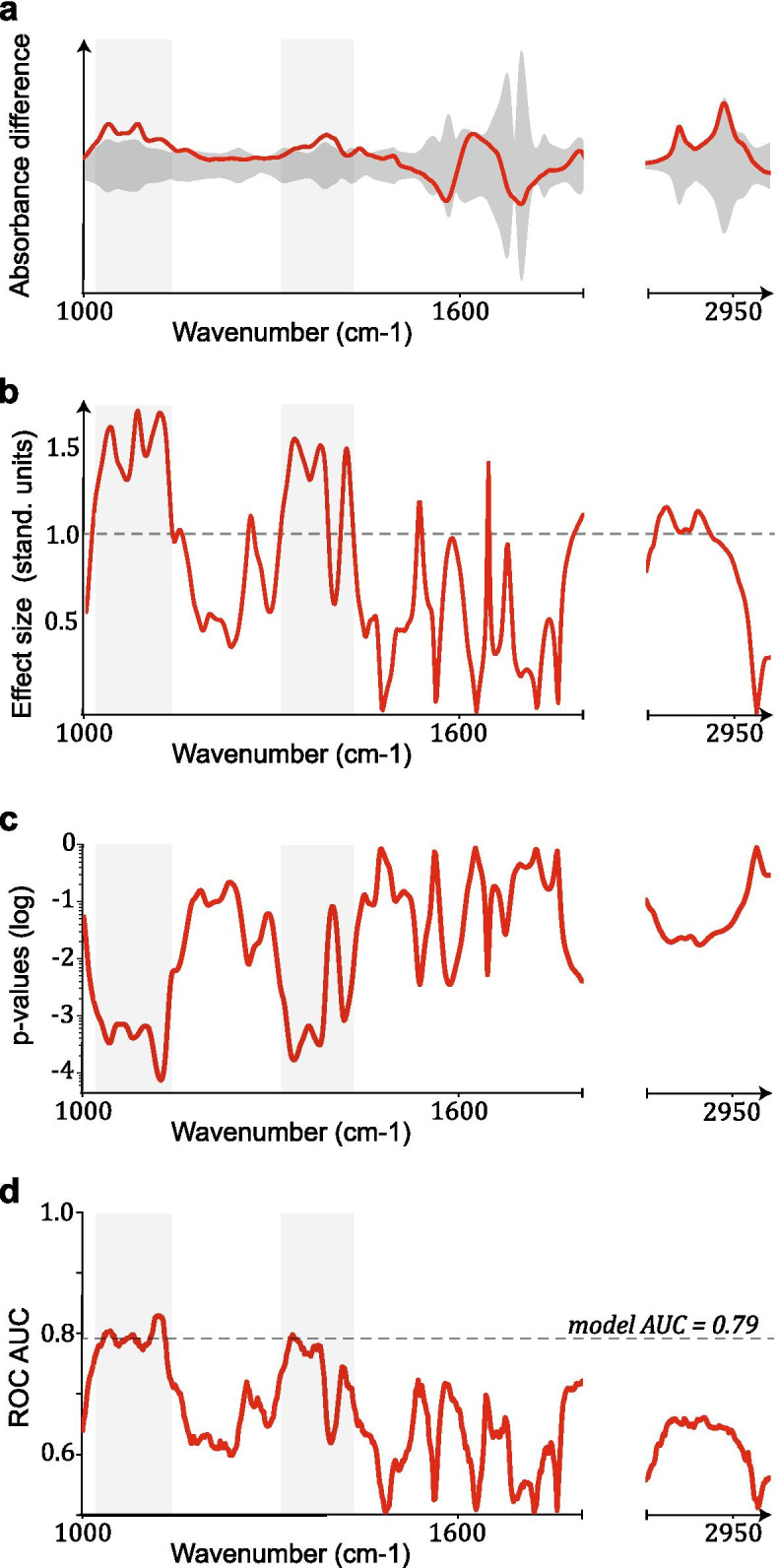
Spectral features. **a** Mean absorbance difference per wavenumber between cases and references (differential fingerprint) **b** Effect size per wavenumber. This quantity is known as the Cohen’s d in signal detection theory and corresponds to the standardized difference between the mean absorbance of the cases and references. The dashed line indicates effect size of one standard deviation. **c** *P*-values per wavenumber, by performing local two-sided t-tests. **d** ROC AUC extracted by the Mann-Whitney U-test. The dashed line corresponds to the AUC value of the trained SVM model. The shaded rectangular areas, in all panels, indicate spectral regions where highly-significant differences have been identified

In order to understand infrared spectral information responsible for BC identification, we have evaluated the infrared spectral signatures that are relevant for distinguishing breast cancer cases from the reference, control individuals. For this purpose, we evaluated the differential fingerprints that we defined as the difference between the mean IMF of the case cohort and that of the reference cohort (Fig. 2a). This quantity, when compared to the standard deviation of the reference group (shaded area in Fig. 2a), reveals the locations along the spectrum for which the difference between the means of the two groups is larger than the sample standard deviation. These differences become even more apparent in Fig. 2b, which depicts the effect size, defined as the differential fingerprint divided by the standard deviation of the reference group. We reveal that at specific spectral locations, the effect size exceeds the barrier of one standard deviation, indicating potentially significant differences between the sample means of the two distributions.

To evaluate the statistical significance of the differences detected in latter analysis when comparing two groups of data, we additionally determined the *p*-value per wavenumber by performing two-sided t-tests. Importantly, we find that *p*-values of highest significance, as low as 10^*−* 4^, are observed in the spectral regions that directly correspond to large effect size (Fig. 2c). Moreover, to further examine the comparison, we calculated the AUC per wavenumber using the U statistic of a Mann-Whitney U test (as described in the Methods section). We observe that the AUC per wavenumber follows a similar pattern as the effect size (Fig. 2d). Interestingly, for the wavenumbers with the lowest p-values and the most significant differences, the single-feature AUC reaches (and in some cases exceeds) the one obtained from the application of the SVM model trained on the entire spectrum (dashed line in Fig. 2d).

The results we provide are the first indication that the presented approach is feasible for the purpose of BC detection and that the predictive power of machine learning can be further leveraged in future analyses requiring larger sample sets. Our presented feasibility evaluation is instrumental for the establishment of a lower bound of the AUC and motivates the collection of larger data and sample sets which shall increase the prediction performance and capacity of the approach.

### Efficiency of breast cancer detection at different stages of malignancy

Cancer detection is challenged by the enormous biological and clinical complexity of cancer, and detection is further complicated by the significant intra-tumor heterogeneity as well as by the impact of the tumour micro-environment [33]. To evaluate whether the blood-based IMFs are sufficiently sensitive to detect tumors at different stages of progression, we first investigated whether the IMF characteristics depend on the stage of the tumor, characterized in terms of clinical TNM (tumor node metastasis) staging [34]. For this purpose, we split the BC cases into two groups and compared them separately with the non-symptomatic, reference individuals. The first group corresponds to the non-metastatic (M0) patients (stages I, II, III) and the second group to metastatic (M1) patients at tumor stage IV. The characteristics of the two groups are shown in Table 2.

**Table 2:** Breakdown of cases in terms of cancer staging

Covariates	M0 cases (*n* = 16)	M1 cases (*n* = 10)
Age in years (mean *±* std)	48 *±* 10	50 *±* 7
BMI in kg/m^2^ (mean *±* std)	29 *±* 6	29 *±* 6
Gender (% female)	100	100

**Fig. 3:**
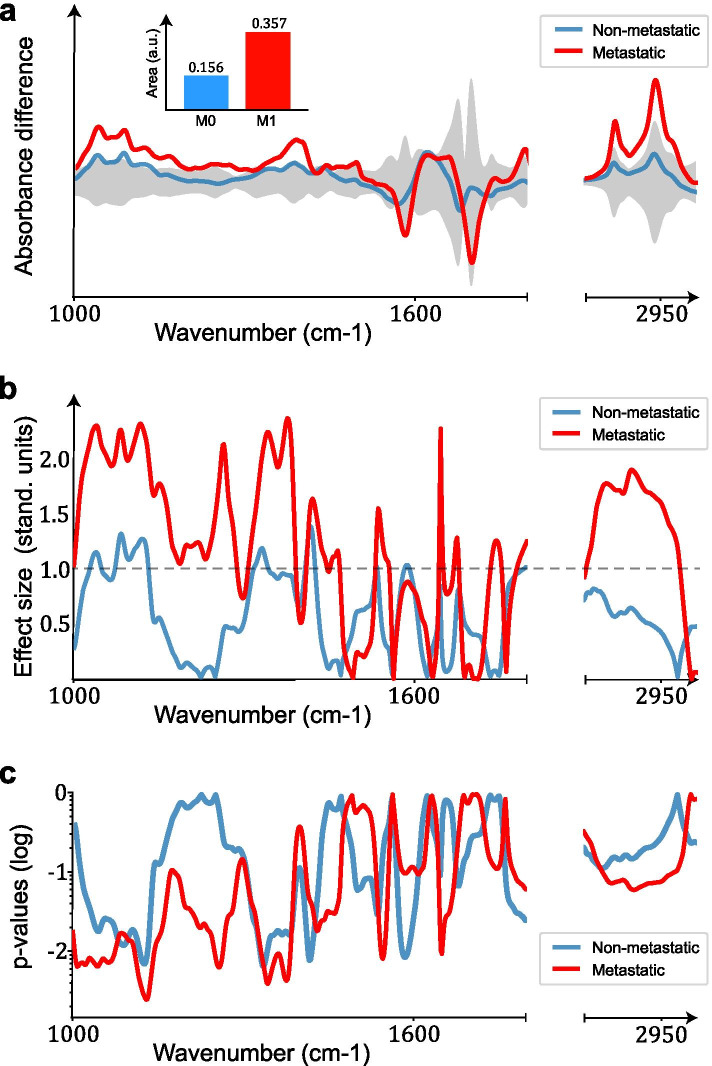
Tumor staging. **a** Mean absorbance difference per wavenumber (differential fingerprint) between cases and references, for metastatic and non-metastatic patients. The inset shows the relative sizes of the area enclosed by the two differential fingerprints. **b** Effect size per wavenumber, for metastatic and non-metastatic patients. The dashed line indicates effect size of one standard deviation. **c** *P*-values per wavenumber, by performing local two-sided t-tests

Panels (a) and (b) in Fig. 3 depict the differential fingerprints, and the effect size per wavenumber and the area enclosed by the differential fingerprint, for each case group compared separately to the controls. *P*-values lower than 10^*−* 2^ are observed in the spectral regions that correspond to large effect size (3 c). Altogether, we observe that the differences between cases and references are much more pronounced across the entire shown spectral range for the metastatic cases with stage IV tumours.

## Discussion

This study provides the first indication that the molecular differences of blood plasma between reference individuals and matched therapy-naive breast cancer females have the potential to be detected with infrared fingerprinting of crude, native liquid plasma. Although previous studies on BC detection have yielded fairly high classification efficiencies [28], they have used dried sera samples, which is known for its limitations.

As a novelty of the approach, here we showed that similar efficiencies can be achieved using measurements of liquid plasma directly. This is advantageous, especially as native plasma sample measurements are more reproducible, require only minimal sample processing and are thus more time efficient, while not leading to known artifacts such as the so called “coffee-ring effect” [35].

This work provides an assessment of the feasibility of infrared molecular probing for breast cancer detection by implementing robust matching that eliminates age and BMI as possible confounding factors. Although the matching excluded a lot of collected data, it is set such that it provides unambiguous assessment of the suitability of the approach. Albeit being very promising, the results of this study need to be further extended and evaluated in larger populations, as we could not involve many of the collected samples into our final investigation, and furthermore, samples from multiple clinical sites need to be further investigated. The findings of this study indicate that the predictive power of machine learning can be further leveraged in future analyses requiring larger sample sets. Our presented feasibility evaluation is instrumental for the establishment of a lower bound of the AUC and motivates the collection of larger data and sample sets which shall increase the prediction performance and capacity of the approach. Importantly, given the ease and stability of FTIR operational workflows to probe bulk fluid plasma, the approach presented here is robust and reproducible [22] and shall be extendable to larger cohorts in a straightforward way to any given population.

Given that this clinical study has been performed on a population enrolling women living in Saudi Arabia, it will be important to evaluate whether blood-based infrared fingerprinting - as a new phenotyping modality - is in position to detect breast-cancer-specific signals independent of different genetic backgrounds and lifestyles. In particular, it will be essential to investigate whether the presented approach could possibly contribute to lowering the rate of false positive outcomes from current screening programs, to possibly provide an additional new approach to be combined with mammography.

Overall, we find a consistent pattern of infrared spectral changes encoded in the IMFs which is more pronounced in the case of more progressed BC stages (either larger tumour volume, or metastatic spread). Although performed within a limited study setting, these findings suggest that the information retrieved from the measured differences between the IMFs of BC cases and references is connected to cancer-related molecular changes. These changes may be due to larger tumour load leaving a more extensive footprint on the composition of peripheral blood, or to the fact that tumour progression could have caused a higher systemic response, or to a combination of both.

## Conclusions

This is a pilot study applying infrared spectroscopy of liquid blood plasma in combination with machine learning for the detection of cancer, showcased on the example of BC. This approach to BC detection, using liquid biopsies, enabled us to differentiate between patients with BC and non-symptomatic reference individuals with an AUC of 0.79, importantly, prior to any cancer-related therapy. In addition, statistical testing shows that the informative signals, captured by the IMFs, are related to the progression of the disease. This pilot study has been performed on a limited cohort with specific characteristics and thus further studies for validating the results on independently-collected samples are necessary. A large-scale validation study is in progress, and additional studies on the detection of several other tumour types are on the way. If proven for its feasibility, given the ease of technical implementation along with the possibility to be extended to high-throughput populational level, this approach possesses the capability to address currently unmet needs in oncology, and has a potential to contribute to the future of precision medicine. Given the time- and cost-efficiency of the approach, we envisage it to be possibly applied in the initial phase of primary disease diagnostics. The main objective may not be to isolate new biomarker candidate molecules, but to efficiently probe with minimally-invasive liquid biopsies in the first instance, before individuals proceed to further diagnostic approaches (based on gold-standard diagnosis by tissue biopsy/radiology).

## Supplementary Information

**Additional file 1.**

## Data Availability

Anonymized raw datasets are available along with the manuscript. Any additional information and data are available upon reasonable request. The custom code used for the production of the results presented in this manuscript is stored in a persistent repository at the Leibniz Supercomputing Center of the Bavarian Academy of Sciences and Humanities (LRZ), located in Garching, Germany. The code can be shared upon reasonable request.

## Abbreviations

BC: Breast cancer
SVM: Support vector machines
FTIR: Fourier-transform infrared
AUC: Area under the curve
ROC: Receiver operating characteristic
IMFs: Infrared molecular fingerprints
MRI: Magnetic resonance imaging
TNM: Tumor node metastasis
CRA: Clinical research associate
BMI: Body-mass index
M1: Metastatic
M0: Non-metastatic
